# Orlicz-Garling sequence spaces of difference operator and their domination in Orlicz-Lorentz spaces

**DOI:** 10.1186/s13660-018-1621-2

**Published:** 2018-02-07

**Authors:** Charu Sharma, Syed Abdul Mohiuddine, Kuldip Raj, Ali H. Alkhaldi

**Affiliations:** 1 School of Mathematics, Shri Mata Vaishno Devi University, Katra, India; 2 Operator Theory and Applications Research Group, Department of Mathematics, Faculty of Science, King Abdulaziz University, Jeddah, Saudi Arabia; 3 Department of Mathematics, College of Science, King Khalid University, Abha, Saudi Arabia

**Keywords:** 46B25, 46B45, 41A65, Banach space, Lorentz sequence space, Garling sequence space, Orlicz function, Subsymmetric bases, Symmetric bases

## Abstract

We introduce new classes of generalized Orlicz-Garling sequences and Orlicz-Lorentz sequences by using a sequence of Orlicz functions and difference operator. We show that the Orlicz-Garling sequence space admits a unique 1-subsymmetric basis and a 1-dominated block basic sequence in $g(\mathcal{M},\Delta^{(m)}, v, p)$. We also make an effort to prove that every symmetric normalized block Orlicz-Garling sequence dominates an Orlicz-Lorentz sequence. Finally, we study some geometric properties of these spaces and establish some inclusion relations between spaces.

## Introduction and preliminaries

Let $c_{00}$ be the space of all scalar sequences, that is, elements of $\mathbb{R}^{\mathbb{N}}$ or $\mathbb{C}^{N}$ with finitely many nonzero entries. Let *X* and *Y* be infinite-dimensional Banach spaces. If $(x_{k})_{k=1}^{\infty}$ is a basis of the Banach space *X* and $x= \sum_{k=1}^{\infty}a_{k}x_{n}$, then we denote $\operatorname{supp}x= \{k\in\mathbb{N}:a_{k}\neq0\}$, that is, the set of indices corresponding to the nonzero entries of *x*, which is called the support of *x*. When the basis $(x_{k})_{k=1}^{\infty}$ is clear from context, we write $x=(a_{k})_{k=1}^{\infty}$ instead of $x= \sum_{k=1}^{\infty}a_{k}x_{n}$.

Now, suppose that $(x_{k})_{k=1}^{\infty}$ and $(y_{k})_{k=1}^{\infty }$ are sequences in *X* and *Y*, respectively. If there exists $C\in [1, \infty)$ such that

$$\Biggl\Vert \sum_{k=1}^{\infty}a_{k}x_{k} \Biggr\Vert _{X}\leq C \Biggl\Vert \sum _{k=1}^{\infty}a_{k}y_{k} \Biggr\Vert _{Y} $$

for all finitely supported sequences $(a_{k})_{k=1}^{\infty}\in c_{00}$, then $(y_{k})_{k=1}^{\infty}$ *C*-dominates $(x_{k})_{k=1}^{\infty}$, and we can write $(x_{k})_{k=1}^{\infty }\lesssim_{C}(y_{k})_{k=1}^{\infty}$. In case *C* does not matter, we will simply say that $(y_{k})_{k=1}^{\infty}$ dominates $(x_{k})_{k=1}^{\infty}$ and $(x_{k})_{k=1}^{\infty}\lesssim (y_{k})_{k=1}^{\infty}$. If $(x_{k})_{k=1}^{\infty}\lesssim _{C}(y_{k})_{k=1}^{\infty}$ and $(y_{k})_{k=1}^{\infty}\lesssim _{C}(x_{k})_{k=1}^{\infty}$, then $(x_{k})_{k=1}^{\infty}$ and $(y_{k})_{k=1}^{\infty}$ are *C*-equivalent, and we can write it as $(x_{k})_{k=1}^{\infty}\approx_{C}(y_{k})_{k=1}^{\infty}$.

A basic sequence $(x_{k})_{k=1}^{\infty}$ is called subsymmetric just in case it is unconditional and equivalent to each of its subsequences. It is called symmetric whenever it is unconditional and equivalent to each of its permutations. Lindenstrauss and Tzafriri [1] studied the uniform boundedness which means that if $(x_{k})_{k=1}^{\infty}$ is a subsymmetric basic sequence, then there is a uniform constant $C \geq1$ such that if $(x_{k_{n}})_{n=1}^{\infty}$ is any subsequence and $(\varepsilon _{n})_{n=1}^{\infty}$ is any sequence of signs, then $(x_{k})_{k=1}^{\infty}$ is *C*-equivalent to $(\varepsilon _{n}x_{k_{n}})_{n=1}^{\infty}$. In this case, we say that $(x_{k})_{k=1}^{\infty}$ is *C*-subsymmetric. Similarly, if $(x_{k})_{k=1}^{\infty}$ is symmetric, then there is $C \geq1$ such that $(x_{k})_{k=1}^{\infty}$ is *C*-equivalent to each $(\varepsilon _{k}x_{\varrho(k)})_{k=1}^{\infty}$, where *ϱ* is a permutation of $\mathbb{N}$, and in this case, we say that $(x_{k})_{k=1}^{\infty}$ is *C*-symmetric. Note that *C*-symmetry implies *C*-subsymmetry, which in turn implies *C*-unconditionality. In this paper, we use standard facts and notation from Banach spaces and approximation theory. For necessary background, see [1, 2], and references therein. We denote by $\mathbb{F}$ the real or complex field. The canonical basis of $\mathbb{F}$ is denoted by $(e_{k})_{k=1}^{\infty}$, that is, $e_{k}=(\delta _{k,n})_{n=1}^{\infty}$, where $\delta_{k,n}=1$ if $n=k$ and $\delta _{k,n}=0$ if $n\neq k$.

An Orlicz function *M* is a function that is continuous, nondecreasing, and convex with $M(0) = 0$, $M(x)>0$ for $x>0$ and $M(x) \longrightarrow\infty$ as $x \longrightarrow\infty$. An Orlicz function *M* is said to satisfy $\Delta_{2}$-condition if there exists $R > 0$ such that $M(2u) \leq R M(u)$, $u \geq0$.

The study of Orlicz sequence spaces was initiated with a certain specific purpose in Banach space theory. Lindenstrauss and Tzafriri [3] used the idea of an Orlicz function to define the sequence space

$$\ell_{M} = \Biggl\{ x \in w : \sum^{\infty}_{k=1} M \biggl(\frac{ \vert x_{k} \vert }{\rho}\biggr) < \infty \text{ for some } \rho>0\Biggr\} , $$

which is called an Orlicz sequence space. The space $\ell_{M}$ is a Banach space with the norm

$$\Vert x \Vert = \inf\Biggl\{ \rho> 0 : \sum^{\infty}_{k=1} M\biggl(\frac{ \vert x_{k} \vert }{\rho}\biggr) \leq 1\Biggr\} . $$

It is shown in [3] that every Orlicz sequence space $\ell_{M}$ contains a subspace isomorphic to $\ell_{p}$ ($p \geq1$).

A sequence $\mathcal{M} = (M_{k})$ of Orlicz functions is called a *Musielak-Orlicz function* (see [4, 5]). For more detail about sequence spaces, see [6–26], and references therein.

The notion of a difference operator in the sequence spaces was first introduced by Kızmaz [27]. The idea of difference sequence spaces of Kızmaz was further generalized by Et and Çolak [28]. Later, this concept was studied by Bektaş et al. [29] and Et et al. [30]. Now, the difference matrix $\Delta= \delta_{nk}$ defined by

$$\delta_{nk}= \textstyle\begin{cases} (-1)^{n-k} & (n-1\leq k\leq n), \\ 0 & (0\leq k< n-1 \text{ or } n> k). \end{cases} $$

The difference operator of order *m* is defined by $\Delta^{m}: w\rightarrow w$, $(\Delta^{1}x)_{k}= (x_{k}-x_{k-1})$ and $\Delta ^{m}x= (\Delta^{1}x)_{k}\circ(\Delta^{m-1}x)_{k}$ for $m\geq2$.

The triangle matrix $\Delta^{(m)}=\delta^{(m)}_{nk}$ defined by

$$\delta^{(m)}_{nk}= \textstyle\begin{cases} (-1)^{n-k}\big( {\scriptsize\begin{matrix}{} m \cr n-k \end{matrix}} \big) & (\max\{0,n-m\}\leq k\leq n), \\ 0 & (0\leq k< \max\{0, n-m\} \text{ or } n> k) \end{cases} $$

for all $k,n \in\mathbb{N}$ and any fixed $m\in\mathbb{N}$.

The Lorentz sequence space was introduced by Lorentz [31, 32]. This space plays an important role in the theory of Banach spaces, whereas Garling [33] studied, for $v=(k^{-1/2})_{k=1}^{\infty}$, the canonical unit vectors of $g(v, 1)$ from a subsymmetric basic sequence that is not symmetric. Pujara [34] studied $g(v, 2)$ for $v=(k^{-1/2})_{k=1}^{\infty}$ and showed that these spaces are uniformly convex and that their canonical basis is subsymmetric but not symmetric. The space from [35] can be viewed as being constructed from variations of $g(w, 1)$ for various choices of weights and gives a canonical basis that is 1-greedy and subsymmetric but not symmetric. Both the Garling sequence spaces and the Lorentz sequence spaces are defined by taking the completion of $c_{00}$ under the norms $\Vert\cdot\Vert_{g}$ or $\Vert\cdot \Vert_{d}$, respectively. The only difference between these norms is that $\Vert\cdot\Vert_{g}$ is defined by taking a certain supremum over subsequences instead of permutations of sequences as is the case for $\Vert\cdot\Vert_{d}$. Wallis [36] generalized the construction of Garling for each $1\leq p< \infty$ and normalized nonincreasing sequence of positive numbers that exhibited complementably homogeneous Banach Garling space related to the Lorentz sequence space. For more detail about these spaces, see [37–39], and references therein.

Let us consider the set of weights

$$V= \bigl\{ (v_{k})_{k=1}^{\infty}\in c_{0} \setminus l_{1}:1=v_{1}\geq v_{2}>\cdots\geq v_{k}\geq v_{k+1}\geq\dots>0\bigr\} . $$

Let $\mathcal{M}=(M_{k})$ be a sequence of Orlicz functions, and let $v=(v_{k})_{k=1}^{\infty}\in V$ be a weight, that is, a sequence of positive scalars. Then the Orlicz-Garling sequence space is defined as the Banach space consisting of all scalar sequences $A= (a_{k})_{k=1}^{\infty}$ such that

$$\begin{aligned} &\Vert A \Vert _{g(\mathcal{M},\Delta^{(m)},v,p)}\\ &\quad = \inf\Biggl\{ \rho>0: \sup _{\psi\in\mathcal{O}}\Biggl(\sum_{k=1}^{\infty} \biggl(M_{k}\biggl(\frac{ \vert \Delta^{(m)}a_{\psi(k)} \vert ^{p}v_{k}}{\rho}\biggr)\biggr)\Biggr)^{\frac{1}{p}} \leq1 \text{ for some } \rho>0\Biggr\} , \end{aligned} $$

where $\mathcal{O}$ denotes the set of all increasing functions from $\mathbb{N}$ to $\mathbb{N}$. We will assume that *v* is normalized, that is, $v_{1}=1$. We consider the normed space $g(\mathcal{M},\Delta ^{(m)},v, p)= \{A= (a_{k})_{k=1}^{\infty}: \Vert A \Vert_{g(\mathcal {M},\Delta^{(m)},v,p,)}<\infty\}$.

Let Π denote the set of permutations on $\mathbb{N}$, and let $\mathcal{M}=(M_{k})$ be a sequence of Orlicz functions. Then, for any $1\leq p< \infty$ and $v\in V$, we define the Orlicz-Lorentz sequence space $d(\mathcal{M},\Delta^{(m)},v,p)$ as the completion of $c_{00}$ under the norm $\Vert\cdot\Vert_{d(\mathcal{M},\Delta ^{(m)},v,p)}$ defined by

$$\begin{aligned} &\Vert A \Vert _{d(\mathcal{M},\Delta^{(m)},v,p)}\\ &\quad = \inf\Biggl\{ \rho>0: \sup _{\varrho\in\Pi}\Biggl(\sum_{k=1}^{\infty } \biggl(M_{k}\biggl(\frac{ \vert \Delta^{(m)}a_{\varrho(k)} \vert ^{p}v_{k}}{\rho}\biggr)\biggr)\Biggr)^{\frac{1}{p}} \leq1 \text{ for some } \rho>0\Biggr\} . \end{aligned} $$

We consider the normed space $d(\mathcal{M},\Delta^{(m)},v, p)= \{A= (a_{k})_{k=1}^{\infty}: \Vert A \Vert_{d(\mathcal{M},\Delta ^{(m)},v,p,)}<\infty\}$.

The main aim of this paper is to introduce and study some difference Orlicz-Garling sequence spaces and Orlicz-Lorentz sequence spaces. Using the originally developed Orlicz-Lorentz sequence space, we show that the Orlicz-Garling sequence space admits a unique 1-subsymmetric basis. Finally, we discuss some additional geometric properties of $g(\mathcal{M},\Delta^{(m)}, v, p)$ and also establish some inclusion relations between these spaces.

## Main results on $g(\mathcal{M},\Delta ^{(m)}, v, p)$

Given a function *ψ*, we denote by $U(\psi)$ its range. Let $\mathcal{M}=(M_{k})$ be a sequence of Orlicz functions, and let $v=(v_{k})_{k=1}^{\infty}\in V$ be a weight. Let $\mathcal{O}_{A}$ be the set of increasing functions from an integer interval $[1,\ldots,u]\cap \mathbb{N}$ into $\mathbb{N}$. For a given $\psi\in\mathcal {O}_{A}$, we denote by $u(\psi)$ the largest integer in its domain; a function in $\mathcal{O}_{A}$ is univocally determined by its range. For $A=(a_{k})_{k=1}^{\infty}$, we have

2.1 $$ \begin{aligned}[b] \Vert A \Vert _{g}^{\mathcal{M},\Delta^{(m)},p}&= \inf\Biggl\{ \rho>0: \sup_{\psi\in\mathcal{O}_{A}}\sum _{k=1}^{u(\psi)}\biggl(M_{k}\biggl( \frac{ \vert \Delta^{(m)}a_{\psi(k)} \vert ^{p}v_{k}}{\rho}\biggr)\biggr)\leq1 \\ &\quad \text{ for some } \rho>0\Biggr\} . \end{aligned} $$

A Banach space with an unconditional basis is said to have a unique unconditional if any two seminormalized unconditional bases of *X* are equivalent.

Now we define the weak Orlicz-Lorentz sequence space $d_{\infty }(\mathcal{M},\Delta^{(m)}, v, p)$ for a sequence of Orlicz functions $\mathcal{M}=(M_{k})$. For $v\in V$ and $1\leq p< \infty$, it consists of all sequences $A=(a_{k})_{k=1}^{\infty}\in c_{0}$ such that

$$\Vert A \Vert _{d_{\infty}(\mathcal{M},\Delta ^{(m)},v,p)}= \inf\Biggl\{ \rho>0: \sup_{k} \Biggl(\sum_{n=1}^{k}\biggl(M_{k} \biggl(\frac{ \vert \Delta^{(m)}a_{k}^{*} \vert v_{n}}{\rho}\biggr)\biggr)\Biggr)^{\frac{1}{p}}\leq1 \text{ for some } \rho>0\Biggr\} , $$

where $(a_{k}^{*})_{k=1}^{\infty}$ denotes the decreasing rearrangement of *A*.

Let $(x_{k})_{k=1}^{\infty}$ be a basis for a Banach space *X*, and let $D= \sum_{k=1}^{\infty}b_{k}x_{k}$ be a vector in *X*. For any $A= \sum_{k=1}^{\infty}a_{k}x_{k}\in X$, it can be written as $A\prec D$ if

(i) $\operatorname{supp}(A)\subseteq \operatorname{supp}(D)$,
(ii) $a_{k}=b_{k}$ for all $k\in \operatorname{supp}(A)$, and
(iii) $\Vert A \Vert= \Vert D \Vert$.

We can say that *D* is minimal in *X* if it is minimal in *X* equipped with the partial order ≺, that is, if $A\prec D$ implies $A=D$. If *D* is supported with respect to a basis $(x_{k})_{k=1}^{\infty}$, then there exists a minimal $A\prec D$.

A block basic sequence of a basic sequence $(x_{k})_{k=1}^{\infty}$ in a Banach space *X* is a sequence $(y_{k})_{k=1}^{\infty}$ of nonzero vectors of the form

$$y_{k}= \sum_{j=p_{k}}^{p_{k+1}-1}a_{j}x_{j} $$

for some increasing sequence of integers $(p_{k})_{k=1}^{\infty}$ with $p_{1}=1$ and some $(a_{k})_{k=1}^{\infty}\in\mathbb{F}^{\mathbb{N}}$.

### Theorem 2.1

*Let* $\mathcal{M}=(M_{k})$ *be a sequence of Orlicz functions*, *and let* $v=(v_{k})_{k=1}^{\infty}\in V$ *be a weight*. *For* $1\leq p< \infty$, *we have* $l_{p}\subsetneq d(\mathcal{M},\Delta ^{(m)}, v, p) \subseteq g(\mathcal{M},\Delta^{(m)} v, p) \subseteq d_{\infty}(\mathcal{M},\Delta^{(m)}, v, p)$, *with norm inclusions*.

### Proof

It is clear that $l_{p}\subsetneq d(\mathcal{M}, \Delta ^{(m)},v, p)$. Let $\psi\in \mathcal{O}_{A}$, $A=(a_{k})_{k=1}^{\infty}\in\mathbb{F}^{\mathbb{N}}$, and $\varrho \in\Pi$ extending *ψ*. Then we have

$$ \begin{aligned} \sum_{k=1}^{t(\psi)}\biggl(M_{k} \biggl(\frac{ \vert \Delta^{(m)}a_{\psi (k)} \vert ^{p}v_{k}}{\rho}\biggr)\biggr) &= \sum_{k=1}^{t(\psi )} \biggl(M_{k}\biggl(\frac{ \vert \Delta^{(m)}a_{\varrho(k)} \vert ^{p}v_{k}}{\rho}\biggr)\biggr) \\ &\leq \sum_{k=1}^{\infty}\biggl(M_{k} \biggl(\frac{ \vert \Delta ^{(m)}a_{\varrho(k)} \vert ^{p}v_{k}}{\rho}\biggr)\biggr) \\ &\leq \Vert A \Vert _{d(\mathcal{M},\Delta^{(m)},v,p)}. \end{aligned} $$

Taking the supremum on *ψ*, we get $\Vert A \Vert_{g(\mathcal {M},\Delta^{(m)},v,p)}\leq \Vert A \Vert_{d(\mathcal{M},\Delta ^{(m)},v,p)}$. Let $A= (a_{k})_{k=1}^{\infty}\in c_{00}$. Given $t(\psi)=k$ and $|a_{\psi(n)}|\geq|a_{k}^{*}|$ for $1\leq n\leq t(\psi)=k$, there exists $\psi\in\mathcal{O}_{A}$ such that, for a given $k\in\mathbb{N}$, we have

$$\begin{aligned} \Biggl(\sum_{n=1}^{k} \biggl(M_{k}\biggl(\frac{ \vert \Delta^{(m)}a_{k}^{*} \vert v_{n}}{\rho}\biggr)\biggr) \Biggr)^{\frac{1}{p}} \leq& \Biggl(\sum_{n=1}^{k} \biggl(M_{k}\biggl(\frac{ \vert \Delta^{(m)}a_{\varrho(n)} \vert ^{p}v_{n}}{\rho}\biggr)\biggr) \Biggr)^{\frac{1}{p}}\leq \Vert A \Vert _{d(\mathcal{M},\Delta^{(m)}, v, p)}. \end{aligned}$$

Therefore, $\Vert A \Vert_{d_{\infty}(\mathcal{M}, \Delta^{(m)}, v, p)}\leq \Vert A \Vert_{g(\mathcal{M}, \Delta^{(m)}, v, p)}$, which concludes the proof. □

Let us define some linear functions as follows:

(i) For a given sequence of signs $\varepsilon= (\varepsilon_{k})_{k=1}^{\infty}$, we define the linear mapping $T_{\varepsilon}:\mathbb{F}^{\mathbb{N}}\rightarrow\mathbb {F}^{\mathbb{N}}$ by $T_{\varepsilon}((a_{k})_{k=1}^{\infty})= (\varepsilon_{k}a_{k})_{k=1}^{\infty}$.
(ii) The coordinate projection on $B\subseteq\mathbb{N}$ is defined by $P_{B}: \mathbb{F}^{\mathbb{N}}\rightarrow\mathbb {F}^{\mathbb{N}}$, $P_{B}((a_{k})_{k=1}^{\infty})= (\mu _{k}a_{k})_{k=1}^{\infty}$, where
  $$\mu_{k}= \textstyle\begin{cases} 1 & \text{if } k\in B, \\ 0 & \text{if } n\notin B. \end{cases} $$
(iii) Given $\psi\in\mathcal{O}$, we define the linear mapping $S_{\psi}: \mathbb{F}^{\mathbb{N}}\rightarrow\mathbb {F}^{\mathbb{N}}$ by $S_{\psi}((a_{k})_{k=1}^{\infty})= (a_{\psi (k)})_{k=1}^{\infty}$, and
(iv) we define the linear function $R_{\psi}: \mathbb {F}^{\mathbb{N}}\rightarrow\mathbb{F}^{\mathbb{N}}$ by $R_{\psi }((a_{k})_{k=1}^{\infty})= (b_{k})_{k=1}^{\infty}$, where
  $$b_{k}= \textstyle\begin{cases} a_{n} & \text{if } k=\psi(n), \\ 0 & \text{if } k\notin\psi(n). \end{cases} $$

### Remark 2.2

Given $k\in\mathbb{N}$ and $\psi\in\mathcal{O}$, we have $R_{\psi}\circ S_{\psi}= \mathit{Id}_{\mathbb{F}^{\mathbb{N}}}$ and $S_{\psi}(e_{k})=e_{\psi(k)}$.

### Theorem 2.3

*Let* $\mathcal{M}=(M_{k})$ *be a sequence of Orlicz functions*, *let* $v=(v_{k})_{k=1}^{\infty}\in V$ *be a weight*, *and let* $1\leq p< \infty$. *Let* $\varepsilon= (\varepsilon_{k})_{k=1}^{\infty }$ *be a sequence of signs*. *Let* *B* *be a subset of* $\mathbb{N}$, *and let* *ψ* *be a linear map in* $\mathcal{O}$. *Then we have the following*:

(i) $T_{\varepsilon}$ *and* $P_{B}$ *are norm*-*one operators from* $g(\mathcal{M},\Delta^{(m)}, v, p)$ *into* $g(\mathcal{M},\Delta ^{(m)}, v, p)$.
(ii) $S_{\psi}$ *and* $R_{\psi}$ *are norm*-*one operators from* $g(\mathcal{M},\Delta^{(m)}, v, p)$ *into* $g(\mathcal{M},\Delta ^{(m)}, v, p)$.
(iii) $S_{\psi}$ *is an isometry from* $g(\mathcal {M},\Delta^{(m)}, v, p)$ *into* $g(\mathcal{M},\Delta^{(m)}, v, p)$.
(iv) *The standard unit vectors form a* 1-*subsymmetric basic sequence in* $g(\mathcal{M},\Delta^{(m)}, v, p)$.

### Proof

The proof of (i) is clear since it is a consequence of (ii) and Remark 2.2. (iv) is a consequence of (i), (iii), and Remark 2.2. Therefore, we only prove (ii). Let $A=(a_{k})_{k=1}^{\infty}\in\mathbb{F}^{N}$. It is clear that $\Vert T_{\varepsilon}(A) \Vert_{g(\mathcal{M},\Delta^{(m)}, v, p)}= \Vert A \Vert _{g(\mathcal{M},\Delta^{(m)}, v, p)}$ and $\Vert P_{B}(A) \Vert _{g(\mathcal{M},\Delta^{(m)}, v, p)}= \Vert A \Vert_{g(\mathcal {M},\Delta^{(m)}, v, p)}$. Since $\psi\circ\varphi\in\mathcal {O}$, we have

$$\begin{aligned} \bigl\Vert S_{\psi}(A) \bigr\Vert _{g}^{\mathcal{M},\Delta^{(m)} p}&=\inf\Biggl\{ \rho>0: \sup_{\varphi\in\mathcal{O}} \sum_{k=1}^{\infty}\biggl(M_{k}\biggl( \frac{ \vert \Delta^{(m)}a_{\psi (\varphi(k))} \vert ^{p}v_{k}}{\rho}\biggr)\biggr)\leq1 \text{ for some } \rho>0\Biggr\} \\ &\leq \Vert A \Vert _{g(\mathcal{M},\Delta^{(m)},v, p)}. \end{aligned} $$

Let us take $\varphi\in\mathcal{O}$ and $R_{\psi}(A)= (b_{k})_{k=1}^{\infty}$. As we know, $\psi^{-1}\circ\varphi$ is an increasing function from a set $B\subseteq\mathbb{N}$ to $\mathbb {N}$. Let $\phi: L\rightarrow B$ be increasing and bijective, where $L= \{k\in\mathbb{N}: k\leq|B|\}$. Since $\xi: \psi^{-1}\circ \varphi\circ\phi\in\mathcal{O}\cup\mathcal{O}_{A}$ and $k\leq \phi(k)$ for all $k\in L$, we have

$$\begin{aligned} \sum_{k=1}^{\infty} \biggl(M_{k}\biggl(\frac{ \vert \Delta^{(m)}b_{\psi (\varphi(k))} \vert ^{p}v_{k}}{\rho}\biggr)\biggr) =& \sum _{k\in B}^{\infty}\biggl(M_{k}\biggl( \frac{ \vert \Delta^{(m)}a_{\psi^{-1}\circ \varphi(k)} \vert ^{p}v_{k}}{\rho}\biggr)\biggr) \\ =& \sum_{k\in L}^{\infty}\biggl(M_{k} \biggl(\frac{ \vert \Delta ^{(m)}a_{\xi(k)} \vert ^{p}v_{\phi(k)}}{\rho}\biggr)\biggr) \\ \leq& \sum_{k\in L}^{\infty}\biggl(M_{k} \biggl(\frac{ \vert \Delta ^{(m)}a_{\xi(k)} \vert ^{p}v_{k}}{\rho}\biggr)\biggr)\leq \Vert A \Vert _{g(\mathcal{M},\Delta^{(m)},v, p)}. \end{aligned}$$

Taking the supremum on *φ*, we get $\Vert R_{\psi}(A) \Vert _{g(\mathcal{M},\Delta^{(m)}, v, p)}\leq \Vert A \Vert_{g(\mathcal {M},\Delta^{(m)}, v, p)}$. □

### Theorem 2.4

*Let* $\mathcal{M}=(M_{k})$ *be a sequence of Orlicz functions*, *let* $v=(v_{k})_{k=1}^{\infty}\in V$ *be a weight*, *and let* $1\leq p< \infty$. *Suppose* $A\in c_{00}$ *is minimal in* $g(\mathcal {M},\Delta^{(m)}, v, p)$ *with respect to the canonical basis*. *Then*

2.2 $$ \begin{aligned}[b] \Vert A \Vert _{g(\mathcal{M},\Delta^{(m)},v, p)} &= \inf\Biggl\{ \rho>0:\Biggl(\sum_{k=1}^{u(\varphi)} \biggl(M_{k}\biggl(\frac{ \vert \Delta^{(m)}a_{\varphi(k)} \vert ^{p}v_{k}}{\rho }\biggr)\biggr) \Biggr)^{\frac{1}{p}}\leq1, \\ &\quad \textit{ for some } \rho>0\Biggr\} , \end{aligned} $$

*where* $\varphi\in\mathcal{O}_{A}$ *is determined by* $U(\varphi)=\operatorname{supp}(A)$.

### Proof

For given $\psi\in\mathcal{O}_{A}$, let *φ* be the function in $\mathcal{O}_{A}$ determined by $U(\varphi)=U(\psi)\cap \operatorname{supp}(A)$. Letting *ϕ* be the inverse function of *ψ* restricted to $U(\varphi)$, we have

$$\begin{aligned} \sum_{k=1}^{u(\psi)} \biggl(M_{k}\biggl(\frac{ \vert \Delta^{(m)}a_{\psi (k)} \vert ^{p}v_{k}}{\rho}\biggr)\biggr) =& \sum _{k\in U(\varphi )}\biggl(M_{k}\biggl(\frac{ \vert \Delta^{(m)}a_{k} \vert ^{p}v_{\phi(k)}}{\rho}\biggr) \biggr) \\ \leq& \sum_{k\in U(\varphi)}\biggl(M_{k}\biggl( \frac{ \vert \Delta ^{(m)}a_{k} \vert ^{p}v_{\varphi^{-1}(k)}}{\rho}\biggr)\biggr) \\ =&\sum_{k\in u(\varphi)}\biggl(M_{k}\biggl( \frac{ \vert \Delta ^{(m)}a_{\varphi(k)} \vert ^{p}v_{k}}{\rho}\biggr)\biggr). \end{aligned}$$

Therefore, we can restrict it to $\{\psi\in\mathcal{O}_{A}: U(\psi )\subseteq \operatorname{supp}(A)\}$, so that supremum (2.1) is attained. Let $\varphi\in\mathcal{O}_{A}$ with $U(\varphi)\subseteq \operatorname{supp}(A)$, and let *H* be the projection of *A* onto $U(\varphi)$. Then, we have

$$\begin{aligned} \Vert A \Vert _{g}^{\mathcal{M},\Delta^{(m)}, p} =& \sum _{k\in u(\varphi)}\biggl(M_{k}\biggl(\frac{ \vert \Delta ^{(m)}a_{\varphi(k)} \vert ^{p}v_{k}}{\rho}\biggr) \biggr) \\ \leq& \Vert H \Vert _{g}^{\mathcal{M},\Delta^{(m)}, p}. \end{aligned}$$

Thus, $H=A$ and $U(\varphi)= \operatorname{supp}(A)$. □

### Theorem 2.5

*Let* $\mathcal{M}=(M_{k})$ *be a sequence of Orlicz functions*, *let* $v=(v_{k})_{k=1}^{\infty}\in V$ *be a weight*, *and let* $1\leq p< \infty$. *Every normalized block basic sequence of the canonical basis of* $g(\mathcal{M},\Delta^{(m)}, v, p)$ *is* 1-*dominated by the canonical basis of* $l_{p}$.

### Proof

Let a normalized block basic sequence be $y_{k}= \sum_{j=p_{k}}^{p_{k+1}-1}a_{j}g_{j}$, $k\in \mathbb{N}$. Let us define $k=k(j)\in\mathbb{N}$ by $p_{k}\leq j\leq p_{k+1}-1$ for $j\in\mathbb{N}$. Let $(c_{j})_{j=1}^{\infty}= \sum_{k=1}^{\infty}b_{k}y_{k}$ and $(b_{k})_{k=1}^{\infty}\in c_{00}$. Let $\psi\in\mathcal{O}$, and let for each $k\in\mathbb{N}$, $L_{k}= \{j\in\mathbb{N}: p_{k}\leq \psi(j)\leq p_{k+1}-1\}$ be an increasing interval. Then, for some $s_{k}$, $u_{k}\in\mathbb{N}\cup\{0\}$, $L_{k}= \{j\in\mathbb{N}: 1+s_{k}\leq i\leq s_{k}+u_{k}\}$. We obtain

$$ \begin{aligned} &\sum_{k=1}^{\infty} \biggl(M_{k}\biggl(\frac{ \vert \Delta^{(m)}c_{\psi (k)} \vert ^{p}v_{k}}{\rho}\biggr)\biggr)\\ &\quad \leq \sum _{k=1}^{\infty }\biggl(M_{k}\biggl( \frac{ \vert \Delta^{(m)}b_{k} \vert ^{p}}{\rho }\biggr)\biggr)\sum_{j\in L_{k}} \biggl(M_{k}\biggl(\frac{ \vert \Delta^{(m)}a_{\psi (j)} \vert ^{p}v_{j}}{\rho}\biggr)\biggr) \\ &\quad = \sum_{k=1}^{\infty}\biggl(M_{k} \biggl(\frac{ \vert \Delta^{(m)}b_{k} \vert ^{p}}{\rho}\biggr)\biggr)\sum_{i=1}^{u_{k}} \biggl(M_{k}\biggl(\frac{ \vert \Delta^{(m)}a_{\psi(i+s_{k})} \vert ^{p}v_{i+s_{k}}}{\rho}\biggr)\biggr) \\ &\quad \leq \sum_{k=1}^{\infty}\biggl(M_{k} \biggl(\frac{ \vert \Delta ^{(m)}b_{k} \vert ^{p}}{\rho}\biggr)\biggr)\sum_{i=1}^{u_{k}} \biggl(M_{k}\biggl(\frac { \vert \Delta^{(m)}a_{\psi(i+s_{k})} \vert ^{p}v_{i}}{\rho}\biggr)\biggr) \\ &\quad \leq \sum_{k=1}^{\infty}\biggl(M_{k} \biggl(\frac{ \vert \Delta ^{(m)}b_{k} \vert ^{p}}{\rho}\biggr)\biggr) \Vert y_{k} \Vert _{g}^{\mathcal{M},\Delta^{(m)}, p} \\ &\quad = \sum_{k=1}^{\infty}\biggl(M_{k} \biggl(\frac{ \vert \Delta^{(m)}b_{k} \vert ^{p}}{\rho}\biggr)\biggr). \end{aligned} $$

Taking the supremum on *ψ*, we get the following result. □

### Theorem 2.6

*Let* $\mathcal{M}=(M_{k})$ *be a sequence of Orlicz functions*, *let* $v=(v_{k})_{k=1}^{\infty}\in V$ *be a weight*, *and let* $1\leq p< \infty$. *Let* $(g_{k})_{k=1}^{\infty}$ *denote the canonical basis of* $g(\mathcal{M},\Delta^{(m)}, v, p)$. *Suppose that* $y_{k}= \sum_{j=p_{k}}^{p_{k+1}-1}a_{j}g_{j}$, $k\in \mathbb{N}$, *forms a block basic sequence in* $g(\mathcal{M},\Delta ^{(m)}, v, p)$. *Then there exists another block basic sequence formed by* $\hat{y}_{k}= \sum_{q=\hat{p}_{k}}^{\hat {p}_{k+1}-1}a_{j_{q}}g_{q}$, $k\in\mathbb{N}$, *such that*

(i) $a_{j_{q}}\neq0$ *for* $q\in\mathbb{N}$;
(ii) $\hat{p}_{1}=1$;
(iii) $(j_{q})_{q=\hat{p}_{k}}^{\hat{p}_{k+1}-1}$ *is a subsequence of* $(j)_{j=p_{k}}^{p_{k+1}-1}$ *for each* $k\in\mathbb {N}$;
(iv) $$\begin{aligned} \Vert y_{k} \Vert _{g(\mathcal{M},\Delta^{(m)},v, p)}&= \Vert \hat{y}_{k} \Vert _{g(\mathcal{M},\Delta^{(m)},v, p)}\\ & = \inf\Biggl\{ \rho>0:\Biggl( \sum _{q=\hat{p}_{k}}^{\hat {p}_{k+1}-1}\biggl(M_{k}\biggl( \frac{ \vert \Delta^{(m)}a_{j_{q}} \vert ^{p}v_{q-\hat {p}_{k}+1}}{\rho}\biggr)\biggr)\Biggr)^{\frac{1}{p}} \leq1\\ & \quad \textit{for some } \rho>0 \textit{ and for each } k\in\mathbb{N}\Biggr\} ; \end{aligned} $$
(v) $(\hat{y}_{k})_{k=1}^{\infty}$ *is* 1-*dominated by* $(y_{k})_{k=1}^{\infty}$.

### Proof

Let $\mathcal{M}=(M_{k})$ be a sequence of Orlicz functions, let $v=(v_{k})_{k=1}^{\infty}\in V$ be a weight, and let $1\leq p< \infty$. Now, for each $k\in\mathbb{N}$, we have $B_{k}\subseteq\{p_{k},\ldots,p_{k+1}-1\}$ such that if $m(j)=\#\{u\in B_{k}:u\leq j\}$ for each $p_{k}\leq j\leq p_{k+1}-1$, then

$$\begin{aligned} & \Vert y_{k} \Vert _{g(\mathcal{M},\Delta^{(m)},v, p)} \\ &\quad =\inf \Biggl\{ \rho>0:\Biggl( \sum_{q=\hat{p}_{k}}^{\hat {p}_{k+1}-1} \biggl(M_{k}\biggl(\frac{ \vert \Delta^{(m)}a_{j_{q}} \vert ^{p}v_{q-\hat{p}_{k}+1}}{\rho}\biggr)\biggr) \Biggr)^{\frac{1}{p}}\leq1 \text{ for some } \rho>0\Biggr\} . \end{aligned} $$

We suppose that $a_{j}\neq0$ whenever $j\in B_{k}$ for some $k\in \mathbb{N}$. Therefore, for each $p_{k}\leq j\leq p_{k+1}-1$, we define

$$\tilde{a_{j}}= \textstyle\begin{cases} a_{j} & \text{if } j\in B_{k}, \\ 0 & \text{if } j\notin B_{k}. \end{cases} $$

Let $\tilde{y}_{k} = \sum_{j=p_{k}}^{p_{k+1}-1}\tilde {a}_{j}g_{j}$ in $g(\mathcal{M},\Delta^{(m)} v, p)$ for each $k\in \mathbb{N}$. Then $(\tilde{y}_{k})_{k=1}^{\infty}$ is a normalized block sequence in $g(\mathcal{M},\Delta^{(m)}, v, p)$, which is 1-dominated by $(y_{k})_{k=1}^{\infty}$.

By defining the sequences $(a_{j_{q}})_{q=1}^{\infty}$ and $(\hat {p}_{k})_{k=1}^{\infty}$ we shall construct the block basic sequence $(\hat{y}_{k})_{k=1}^{\infty}$. Suppose $(\tilde {a}_{j_{q}})_{q=1}^{\infty}$ is the subsequence that consists precisely of the nonzero values of $(\tilde{a}_{j})_{j=1}^{\infty}$. It is clear that (i) holds and there exist $1= \hat{p}_{1}<\hat {p}_{2}<\cdots\in\mathbb{N}$ such that (ii) ad (iii) also hold. Now, we find that $\Vert y_{k} \Vert_{g(\mathcal{M},\Delta^{(m)},v, p)}= \Vert\tilde{y}_{k} \Vert_{g(\mathcal{M},\Delta^{(m)},v, p)}= \Vert \hat{y}_{k} \Vert_{g(\mathcal{M},\Delta^{(m)},v, p)}$, which yields (iv). Finally, we have $(\hat{y}_{k})_{k=1}^{\infty}\approx_{1} (\tilde{y}_{k})_{k=1}^{\infty}\lesssim_{1}(y_{k})_{k=1}^{\infty}$ and get (v). □

### Theorem 2.7

*Suppose* $\mathcal{M}=(M_{k})$ *is a sequence of Orlicz functions*, $v=(v_{k})_{k=1}^{\infty}\in V$ *is a weight*, *and* $1\leq p< \infty$. *Let* $(d_{k})_{k=1}^{\infty}$ *denote the canonical basis of* $d(\mathcal{M},\Delta^{(m)}, v, p)$ *and* $(g_{k})_{k=1}^{\infty}$ *denote the canonical basis of* $g(\mathcal{M},\Delta^{(m)}, v, p)$. *Then every symmetric normalized block sequence of* $(g_{k})_{k=1}^{\infty}$ *dominates* $(d_{k})_{k=1}^{\infty}$.

### Proof

Let $\mathcal{M}=(M_{k})$ be a sequence of Orlicz functions, let $v=(v_{k})_{k=1}^{\infty}\in V$ be a weight, and let $1\leq p< \infty$. Take any symmetric normalized block sequence $(y_{k})_{k=1}^{\infty}$ of $(g_{k})_{k=1}^{\infty}$ in $g(\mathcal {M},\Delta^{(m)}, v, p)$. For any finite sequence $(b_{k})_{k=1}^{K}$ for $K\in\mathbb{N}$, we have

$$\begin{aligned} &\Biggl\Vert \sum_{k=1}^{K}b_{k}d_{k} \Biggr\Vert _{d(\mathcal {M},\Delta^{(m)}, v, p)}\\ &\quad = \inf\Biggl\{ \rho>0:\Biggl( \sum _{k=1}^{K}\biggl(M_{k}\biggl( \frac{ \vert \Delta^{(m)}b_{\varrho(k)} \vert ^{p}v_{k}}{\rho}\biggr)\biggr)\Biggr)^{\frac{1}{p}}\leq1 \text{ for some } \rho>0\Biggr\} \end{aligned} $$

for some permutation *ϱ* of $\{1,\ldots,K\}$. However, due to symmetry, we can find that $C\in[1,\infty)$ such that $(y_{k})_{k=1}^{\infty}$ is *C*-equivalent to all its subsequences and *C*-dominates $(g_{k})_{k=1}^{\infty}$. Therefore,

$$\begin{aligned} \Biggl\Vert \sum_{k=1}^{K}b_{k}d_{k} \Biggr\Vert _{d(\mathcal {M},\Delta^{(m)}, v, p)} =& \Biggl(\sum_{k=1}^{K} \biggl(M_{k}\biggl(\frac{ \vert \Delta^{(m)}b_{\varrho(k)} \vert ^{p}v_{k}}{\rho}\biggr)\biggr)\Biggr) ^{\frac{1}{p}} \\ \leq& C^{2} \Biggl\Vert \sum_{k=1}^{K}b_{k}d_{k} \Biggr\Vert _{g(\mathcal{M},\Delta^{(m)} v, p)}. \end{aligned}$$

It gives that $(y_{k})_{k=1}^{\infty}$ $C^{2}$-dominates $(d_{k})_{k=1}^{\infty}$ □

### Theorem 2.8

*Let* $\mathcal{M}=(M_{k})$ *be a sequence of Orlicz functions*, *let* $v=(v_{k})_{k=1}^{\infty}\in V$ *be a weight*, *and let* $1\leq p< \infty$. *Let* $(g_{k})_{k=1}^{\infty}$ *denote the canonical basis of* $g(\mathcal{M},\Delta^{(m)}, v, p)$. *Suppose* $y_{k}= \sum_{j=p_{k}}^{p_{k+1}-1}a_{j}g_{j}$ *forms a normalized block basic sequence and satisfies* $\lim_{j\rightarrow\infty}a_{j}=0$. *Let* $(\hat{y}_{k})_{k=1}^{\infty}$ *be the sequence from Theorem *2.6 *corresponding to* $(y_{k})_{k=1}^{\infty}$. *Then for each* $\varepsilon>0$, *there exists a subsequence* $(y_{k_{n}})_{n=1}^{\infty}$ *that is* $(1+\varepsilon )$-*equivalent to the canonical basis* $(a_{k})_{k=1}^{\infty}$ *such that* $(\hat{y}_{k_{n}})_{n=1}^{\infty}$ *is* $(1 + \varepsilon )$-*complemented in* $g(\mathcal{M},\Delta^{(m)}, v, p)$.

### Proof

The first part of the proof follows that of Lemma 1 in [38]. Let $\varepsilon> 0$, and let $\hat{y}_{k}= \sum_{q=\hat{p}_{k}}^{\hat{p}_{k+1}-1}a_{j_{q}}g_{q}$ form a normalized block basic sequence as defined in Theorem 2.6, and let $\psi\in (0,1)$ be such that $\psi^{-1/p}\leq\psi^{1}<1+\psi$. We claim that there exists a block sequence of the form $z_{k}= \sum_{q=l_{k}}^{l_{k+1}-1}b_{q}g_{q}$ for $n\in\mathbb{N}$ and a subsequence $(\hat{y}_{k_{n}})_{k=1}^{\infty}$ satisfying the following two properties for every $n\in\mathbb{N}$:

(i) The coefficients of $z_{k}$ are the same as the coefficients of $\hat{y}_{k_{n}}$, that is, $l_{n+1}-l_{n}=\hat {p}_{k_{n}+1}-\hat{p}_{k_{n}}$ and $b_{l_{n}+q}=\hat{a}_{j_{\hat {p}_{k_{n}}+1}}$ for $q=0,\ldots,l_{n+1}-l_{n}-1$, and
(ii) $\sum_{q=l_{n}}^{l_{n+1}-1}(M_{q}(\frac {|\Delta^{(m)}b_{q}|^{p}v_{q}}{\rho}))\geq\psi$.

Let $l_{1}=1$ and define $l_{n}$ for some $n\in\mathbb{N}$. Since $v_{k}\rightarrow0$, we have $i\in\mathbb{N}$ such that

$$\sum_{q=i+1}^{i+l_{n}}M\bigl( \vert v_{q} \vert \bigr)< \frac{1-\psi}{2}. $$

Since $a_{j_{q}}\rightarrow0$, we have $\hat{p}_{k+1}-\hat {p}_{k}\rightarrow\infty$. Therefore, for any $h\in\mathbb{N}$ such that $\hat{p}_{h+1}-\hat{p}_{h}> i+l_{n}$, we have $M(|a_{j_{q}}|^{p})< \frac{1-\psi}{2i}$ for all $q\geq\hat{p}_{h}$. Let $l_{n+1}=\hat{p}_{h+1}-\hat{p}_{h}+l_{n}$ and $b_{l_{n}+q}=a_{j_{\hat{p}_{h}-q}}$ for all $q=0,\ldots,l_{n+1}-l_{l}-1$. Since $\hat{y}_{k_{n}}=\hat{y}_{h}$ satisfies (i), by property (iv) of Theorem 2.6 we have

$$1= \Vert \hat{y}_{h} \Vert _{g}^{\mathcal{M}, \Delta ^{(m)}, p}=\inf \Biggl\{ \rho>0: \sum_{q=\hat{p}_{h}}^{\hat {p}_{h+1}-1} \biggl(M_{q}\biggl(\frac{ \vert \Delta^{(m)}a_{j_{q}} \vert ^{p}v_{q-\hat{p}_{h}+1}}{\rho}\biggr)\biggr)\leq1 \text{ for some } \rho >0\Biggr\} , $$

and thus we have

2.3 $$ \begin{aligned}[b] \sum_{q=l_{n}}^{l_{n+1}-1} \biggl(M_{q}\biggl(\frac{ \vert \Delta^{(m)}b_{q} \vert ^{p}v_{q}}{\rho}\biggr)\biggr) &= \sum _{q=\hat {p}_{h}}^{\hat{p}_{h+1}-1}\biggl(M_{q}\biggl( \frac{ \vert \Delta ^{(m)}a_{j_{q}} \vert ^{p}v_{q+l_{n}-\hat{p}_{h}}}{\rho}\biggr)\biggr) \\ &= \sum_{q=\hat{p}_{h}}^{\hat{p}_{h+1}-1}\biggl(M_{q} \biggl(\frac{ \vert \Delta^{(m)}a_{j_{q}} \vert ^{p}v_{q-\hat{p}_{h}+1}}{\rho }\biggr)\biggr) \\ &\quad {}-\sum_{q=\hat{p}_{h}}^{\hat{p}_{h+1}-1} \biggl(M_{q}\biggl(\frac{ \vert \Delta^{(m)}\hat{a}_{q} \vert ^{p}(v_{q-\hat {p}_{h}+1}-v_{q+l_{n}-\hat{p}_{h}})}{\rho}\biggr)\biggr) \\ &= 1- \sum_{q=\hat{p}_{h}}^{\hat{p}_{h+1}-1}\biggl(M_{q} \biggl(\frac{ \vert \Delta^{(m)}a_{j_{q}} \vert ^{p}(v_{q-\hat {p}_{h}+1}-v_{q+l_{n}-\hat{p}_{h}})}{\rho}\biggr)\biggr) \\ &\quad {}-\sum_{q=\hat{p}_{h}+i}^{\hat{p}_{h+1}-1} \biggl(M_{q}\biggl(\frac{ \vert \Delta^{(m)}a_{j_{q}} \vert ^{p}(v_{q-\hat {p}_{h}+1}-v_{q+l_{n}-\hat{p}_{h}})}{\rho}\biggr)\biggr). \end{aligned} $$

Since $(v_{q})_{q=1}^{\infty}$ is nonincreasing with $0< v_{q}\leq1$ for $q\in\mathbb{N}$ and $l_{n}\geq1$, we obtain $0\leq v_{q-\hat {p}_{h}+1}-v_{q+l_{n}-\hat{p}_{h}}<1$ for $q\in\mathbb{N}$. Since there are *i* values between $\hat{p}_{h}$ and $\hat{p}_{h}+i-1$ and also $M(|a_{j_{q}}|^{p})< \frac{1-\psi}{2i}$ for all $q\geq\hat {p}_{h}$, we have

2.4 $$ \begin{aligned}[b] &\sum_{q=\hat{p}_{h}}^{\hat{p}_{h+1}-1} \biggl(M_{q}\biggl(\frac{ \vert \Delta^{(m)}a_{j_{q}} \vert ^{p}(v_{q-\hat {p}_{h}+1}-v_{q+l_{n}-\hat{p}_{h}})}{\rho}\biggr)\biggr) \\ &\quad < \sum_{q=\hat{p}_{h}}^{\hat{p}_{h+1}-1} \biggl(M_{q}\biggl(\frac{ \vert \Delta^{(m)}a_{j_{q}} \vert ^{p}}{\rho}\biggr)\biggr) < \frac{1-\psi}{2i}\sum_{q=\hat{p}_{h}}^{\hat {p}_{h+1}-1}M(1)= \frac{1-\psi}{2}. \end{aligned} $$

Since $M(|a_{j_{q}}|^{p})< \frac{1-\psi}{2i}<1$ for all $q\geq\hat {p}_{h}$, $\sum_{q=i+1}^{i+l_{n}}M(|v_{q}|)< \frac {1-\psi}{2}$, and $\hat{p}_{h+1}+l_{n}-2\geq\hat{p}_{h+1}-1$, we have

2.5 $$ \begin{aligned}[b] &\sum_{q=\hat{p}_{h}+i}^{\hat{p}_{h+1}-1} \biggl(M_{q}\biggl(\frac{ \vert \Delta^{(m)}a_{j_{q}} \vert ^{p}(v_{q-\hat {p}_{h}+1}-v_{q+l_{n}-\hat{p}_{h}})}{\rho}\biggr)\biggr) \\ &\quad < \sum_{q=\hat{p}_{h}+i}^{\hat{p}_{h+1}-1} \biggl(M_{q}\biggl(\frac{v_{q-\hat {p}_{h}+1}-v_{q+l_{n}-\hat{p}_{h}}}{\rho}\biggr)\biggr) \\ &\quad < \sum_{q=\hat{p}_{h}+i}^{\hat{p}_{h+i+l_{n}}-1} \biggl(M_{q}\biggl(\frac {v_{q-\hat{p}_{h}+1}}{\rho}\biggr)\biggr)< \frac{1-\psi}{2}. \end{aligned} $$

Combining (2.3), (2.4), and (2.5), we obtain (ii). This completes the proof of our claim.

Let us prove that a block basic sequence $(z_{k})_{k=1}$ in $g(\mathcal {M},\Delta^{(m)}, v, p)$ dominates the canonical basis $(A_{k})_{k=1}^{\infty}$ for $l_{p}$. For $K\in\mathbb{N}$, let $(c_{k})_{k=1}^{\infty}$ be any finite sequence of scalars. Then we have

$$\begin{aligned} \psi\sum_{k=1}^{K} \biggl(M_{k}\biggl(\frac{ \vert \Delta^{(m)}c_{k} \vert ^{p}}{\rho}\biggr)\biggr) &\leq \sum _{k=1}^{K}\biggl(M_{k}\biggl( \frac{ \vert \Delta^{(m)}c_{k} \vert ^{p}}{\rho}\biggr)\biggr)\sum_{q=l_{k}}^{l_{k+1}-1} \biggl(M_{q}\biggl(\frac{ \vert \Delta^{(m)}b_{q} \vert ^{p}v_{q}}{\rho}\biggr)\biggr) \\ &\leq \Biggl\Vert \sum_{k=1}^{K}c_{k}z_{k} \Biggr\Vert _{g(\mathcal{M},\Delta^{(m)},v,p)}. \end{aligned} $$

Therefore,

$$\Biggl(\sum_{k=1}^{K}\biggl(M_{k} \biggl(\frac{ \vert \Delta^{(m)}c_{k} \vert ^{p}}{\rho}\biggr)\biggr)\Biggr)^{\frac{1}{p}}\leq \psi^{1/p} \Biggl\Vert \sum_{k=1}^{K}c_{k}z_{k} \Biggr\Vert _{g(\mathcal{M},\Delta^{(m)},v,p)}. $$

Thus, $(A_{k})_{k=1}^{\infty}$ is $\psi^{-1/p}$-dominated by $(z_{k})_{k=1}^{\infty}$. On the other hand, $(z_{k})_{k=1}^{\infty}$ is 1-equivalent to $(\hat{y}_{k_{n}})_{k=1}^{\infty}$ by (i) with 1-subsymmetry of $(g_{k})_{k=1}^{\infty}$. By property (v) in Theorem 2.6 the sequence $(\hat{y}_{k})_{k=1}^{\infty}$ is 1-dominated by $(y_{k})_{k=1}^{\infty}$, and by Theorem 2.5 the sequence $(y_{k_{n}})_{n=1}^{\infty}$ is 1-dominated by $(A_{k})_{k=1}^{\infty}$. Now, we have

2.6 $$\begin{aligned} (A_{k})_{k=1}^{\infty} \lesssim_{\psi^{-1/p}}&(z_{k})_{k=1}^{\infty } \thickapprox_{1}(y_{k_{n}})_{n=1}^{\infty} \lesssim_{1} (A_{k})_{k=1}^{\infty}, \end{aligned}$$

and thus $(y_{k_{n}})_{n=1}^{\infty}$ is $\psi^{-1/p}$-equivalent and hence $(1+\varepsilon)$-equivalent to $(A_{k})_{k=1}^{\infty}$.

To show that $(\hat{y}_{k_{n}})_{n=1}^{\infty}$ is $(1+\varepsilon )$-complement in $g(\mathcal{M},\Delta^{(m)},v,p)$, we will follow the proof of Lemma 15 in [39]. Let us define the sequence $\tilde{z}_{n}=\sum_{q=l_{n}}^{l_{n+1}-1}(M_{q}(\frac{|\Delta ^{(m)}b_{q}|^{p}g_{q}}{\rho}))$ for $n\in\mathbb{N}$. Let *P* extend to a linear operator on $(g_{j})_{j=1}^{\infty}$. For each $n\in \mathbb{N}$, we obtain $P\tilde{z}_{n}=P \sum_{j=l_{n}}^{l_{n+1}-1}(M_{j}(\frac{|\Delta^{(m)}b_{j}|^{p}g_{j}}{\rho }))= \hat{z}_{n}$, so that *P* is the projection onto $(\tilde {z}_{n})_{n=1}^{\infty}$. Also by Theorem 2.5, $(\tilde {z}_{n})_{n=1}^{\infty}$ is 1-dominated by $(A_{n})_{n=1}^{\infty }$. Since (ii) holds for each $(c_{j})_{j=1}^{\infty}\in c_{00}$, we get

$$\begin{aligned} \Biggl\Vert P\sum_{j=1}^{\infty}c_{j}g_{j} \Biggr\Vert ^{\mathcal {M},\Delta^{(m)}, p} =& \Biggl\Vert P\sum _{n=1}^{\infty}\sum_{j=l_{n}}^{l_{n+1}-1}c_{j}g_{j} \Biggr\Vert ^{\mathcal{M},\Delta^{(m)}, p} \\ \leq& \psi^{-p} \sum_{n=1}^{\infty} \Biggl\vert \sum_{j=l_{n}}^{l_{n+1}-1} \biggl(M_{j}\biggl(\frac{c_{j} \vert \Delta^{m}b_{j} \vert ^{p-1}v_{j}}{\rho}\biggr)\biggr) \Biggr\vert ^{p}. \end{aligned}$$

Then by the Hölder’s inequality with $\frac{p}{p-1}$ conjugate to *p* and by

$$\Delta^{m}(c_{j}) \bigl\vert \Delta^{m}b_{j} \bigr\vert ^{p-1}v_{j}=\bigl(\Delta^{m}(c_{j})v_{j}^{1/p} \bigr) \bigl( \bigl\vert \Delta ^{m}b_{j} \bigr\vert ^{p-1}v_{j}^{(p-1)/p}\bigr) $$

with $\sum_{j=l_{n}}^{l_{n+1}-1}(M_{j}(\frac{|\Delta ^{(m)}b_{j}|^{p}v_{j}}{\rho}))\leq \Vert z_{n} \Vert^{\mathcal {M},\Delta^{(m)},p}=1$ we have

$$\begin{aligned} \psi^{-p}\sum_{n=1}^{\infty} \Biggl\vert \sum_{j=l_{n}}^{l_{n+1}-1} \biggl(M_{j}\biggl(\frac{\Delta^{m}(c_{j}) \vert\Delta ^{m}b_{j} \vert^{p-1}v_{j}}{\rho}\biggr)\biggr) \Biggr\vert ^{p} \leq& \psi ^{-p}\sum_{n=1}^{\infty} \sum_{j=l_{n}}^{l_{n+1}-1}\biggl(M_{j} \biggl(\frac{ \vert \Delta^{(m)}c_{j} \vert ^{p}v_{j}}{\rho}\biggr)\biggr) \\ \leq& \psi^{-p} \Biggl\Vert \sum_{j=1}^{\infty}c_{j}g_{j} \Biggr\Vert _{g}^{\mathcal{M},\Delta^{(m)},p}. \end{aligned}$$

Therefore, *P* is bounded by $\psi^{-1}$, and thus there is a $\psi ^{-1}$-bounded continuous extension *P̃* in $\mathcal {L}(g(\mathcal{M},\Delta^{(m)},v,p))$, where *P̃* is the projection onto $(\tilde{z}_{n})_{n=1}^{\infty}$. Now define some operators $\Psi_{1}$, $\Theta_{1}$, $\Psi_{2}$, $\Theta_{2}\in \mathcal{L}(g(\mathcal{M},\Delta^{(m)},v,p))$ such that

2.7 $$\begin{aligned} \hat{y}_{k_{n}}\xrightarrow{\Psi_{1}}z_{n} \xrightarrow{\Psi_{2}} \tilde{z}_{n} \quad \text{and}\quad \tilde{z}_{n}\xrightarrow{\Theta _{2}}z_{n} \xrightarrow{\Theta_{1}}\hat{y}_{k_{n}} \end{aligned}$$

for all $n\in\mathbb{N}$. Note that $\Psi_{2}\Theta_{2}=\Theta _{2}\Psi_{2}=I_{g(\mathcal{M},\Delta^{(m)},v,p)}$. By the 1-unconditionality of $(g_{k})_{k=1}^{\infty}$ these are all norm-one operators, and all these satisfy the mappings in (2.7). Let us form the operator

$$\Theta_{1}\Theta_{2}\tilde{P}\Psi_{1} \Psi_{2}\in\mathcal {L}\bigl(g\bigl(\mathcal{M},\Delta^{(m)},v,p \bigr)\bigr). $$

Therefore it is a projection onto $(\hat{y}_{k_{n}})_{n=1}^{\infty}$. This gives the desired result. □
